# Acupuncture for early Parkinson’s disease with mild to moderate depression: a randomized controlled trial protocol with functional MRI

**DOI:** 10.3389/fneur.2024.1457787

**Published:** 2024-10-04

**Authors:** Hongli Xiao, Yashuo Ren, Haosen Yang, Zixi Wang, Zhuohao Li, Yuguo Song, Xiaojia Yuan, Xiaopeng Liu, Peng Chen

**Affiliations:** ^1^Department of Acupuncture and Moxibustion, Beijing Hospital of Traditional Chinese Medicine, Capital Medical University, Beijing, China; ^2^Graduate School, Beijing University of Chinese Medicine, Beijing, China

**Keywords:** acupuncture, depression in Parkinson’s disease, RCT-randomized controlled trial, fMRI, protocol

## Abstract

**Introduction:**

Depression is a common non-motor symptom of Parkinson’s disease (PD), which seriously affects the quality of life of patients with PD. The main clinical treatment method for depression in Parkinson’s disease is medication treatment. However, the medication treatment has a long cycle and many adverse reactions. Acupuncture as a non-pharmacological intervention method, has been widely used in the treatment of patients with Parkinson’s disease and depressive disorders in China. Therefore, the study of acupuncture in the treatment of early Parkinson’s disease with mild to moderate depression has important practical significance.

**Methods and analysis:**

In this randomized, single-blinded, and placebo-controlled study, a total of 88 patients with depression in Parkinson’s disease (DPD) will be randomly allocated to either an acupuncture group or a control group in parallel in a 1:1 allocation ratio. Each group will receive 30 min acupuncture treatments or sham acupuncture treatments, 3 times a week, for 12 weeks, followed by a 36-week follow-up period. The primary outcome is the response rate of the Hamilton Depression Rating Scale-17 at 12 weeks. Data will be collected at baseline, at the end of the 12-week treatment period, and during the 12-week and 36-week follow-up.

**Discussion:**

This study hypothesized that acupuncture may treat DPD by restoring pathological alterations in brain neural activity. The findings will provide scientific evidence for acupuncture in the treatment of early PD with mild to moderate depression.

**Ethics and dissemination:**

This clinical trial has been approved by the Medical Ethics Committee of the Beijing Hospital of Traditional Chinese Medicine (Approval No. 2023BL02-013-01). This trial has been registered with the Chinese Clinical Trials Registry (Registration No. ChiCTR2300069310). The results will be published in a peer-reviewed academic journal.

**Clinical trial registration:**

https://www.chictr.org.cn/, identifier ChiCTR2300069310.

## Introduction

Parkinson’s disease (PD) is a neurodegenerative disease characterized by bradykinesia, rest tremor, and muscle stiffness (1, 2). In addition to symptoms of movement disorders, PD patients also experience various non-motor symptoms, such as depression, rapid eye movement sleep behavior disorder, pain, etc. (3). Approximately 35 to 50% of PD patients will experience depression, which often persists (3, 4).

Depression is a common non-motor symptom that occurs in patients with all stages of PD (5). A meta-study showed that 17% of PD patients in clinical practice suffer from severe depression, 35% have clinically significant depressive symptoms (6), and a portion of patients have suicidal tendencies (7). Depression is one of the biggest factors affecting the quality of life of PD patients, especially female PD patients (8).

In addition, depression is often a sign of other symptoms of PD and has diagnostic value. For early PD patients, depression may be one of the early signs before the onset of motor symptoms (9). For patients with advanced PD, depression may be one of the early signs of more severe non-motor symptoms (dementia, falls, disability) in the later stages (10, 11). Secondly, depression itself can seriously affect the quality of life of Parkinson’s disease patients. Patients with Parkinson’s disease and depression often experience significant negative emotions such as shame, suicidal tendencies, low morale, and despair, and a considerable number of patients die by suicide (12). These negative emotions seriously affect the quality of life (QoL) of Parkinson’s disease patients and significantly impact prognosis (13, 14). Therefore, developing a new treatment plan for Parkinson’s disease with depression has practical significance and can improve the quality of life of patients.

For a long time, research on the tolerance, safety, and efficacy of antidepressants in Parkinson’s disease patients has been very limited (15, 16). A study has found that dopamine agonists may alleviate depression in early Parkinson’s disease (17). Nevertheless, dopamine agonists have side effects on impulse control disorder and sleep dysfunction. Tricyclic antidepressants (TCAs) and selective serotonin reuptake inhibitors (SSRIs) have been subjected to a limited number of trials to assess their therapeutic efficacy in the treatment of DPD. The overall results are positive, but the evidence is not sufficient (15, 18, 19). However, TCA drugs have side effects such as drowsiness, dry mouth, urinary retention, constipation, cognitive impairment, hypotension, and abnormal cardiac conduction, which limit their application in the treatment of DPD. Although SSRI drugs can improve depression in DPD patients, they can worsen their motor symptoms (20). There is a lack of research on SNRI drugs for treating DPD, and there is insufficient evidence to prove the efficacy of such drugs in treating DPD (21). The above-mentioned antidepressants may have a potential tendency to worsen motor or non-motor symptoms in DPD, which encouraged research on non-pharmacological treatment of DPD. Non-pharmacological treatments can be used independently or in conjunction with conventional antidepressants. PD patients often express interest and experience positive emotions towards this treatment strategy (22). Thus, it’s important to research non-pharmacological treatment of DPD.

The efficacy of acupuncture in the treatment of depression has been demonstrated in clinical trials. A randomized controlled trial demonstrated that acupuncture could enhance the effectiveness of antidepressant medications while decreasing their side effects (23). Research shows that acupuncture can alleviate depression by improving neural plasticity, reducing brain inflammation, regulating neuroendocrine, and regulating the expression of brain-derived neurotrophic factors (24–28). Nevertheless, the effect of acupuncture on DPD has not been studied. A study demonstrated the atrophy of the dorsal portion of the anterior cingulate cortex (ACCx) in DPD (29). One experiment in Rat shows acupuncture will increase the activities of glutamatergic neurons in the ACCx and serotoninergic neurons in the DRN, thus significantly alleviating both mechanical allodynia and anxiety-like behaviors (30). Experimental research is needed to verify this hypothesis. Thus, acupuncture may alleviate DPD by changing the brain’s anatomy and function. This hypothesis has to be verified by experimental research.

Pathophysiological studies have found that there are certain differences in the pathogenesis between DPD and primary depression. DPD is a holistic disorder involving the interplay of discrete brain regions rather than a focal structural or functional disorder (15). A meta-study summarized the current research on the functional brain mechanisms of PD with depression and found that its pathogenesis was associated with the cingulate gyrus, supplementary motor function areas, and cerebellar BOLD signaling (25). Therefore, neuropsychological tests combined with functional magnetic resonance imaging (fMRI) may be a better evaluation criterion. Using fMRI to examine the changes of brain function can better observe the clinical efficacy of acupuncture and moxibustion and explore its therapeutic mechanism.

## Methods

### Study design

The methodological framework for this project was developed based on the Consolidated Standards of Reporting Trials (CONSORT) statement for non-pharmacological interventions. The protocol was approved by the Medical Ethics Committee of the Beijing Hospital of Traditional Chinese Medicine (2023BL02-013-01) and is registered with the Chinese Clinical Trial Registry (Registration No. ChiCTR2300069310).

**Figure 1:**
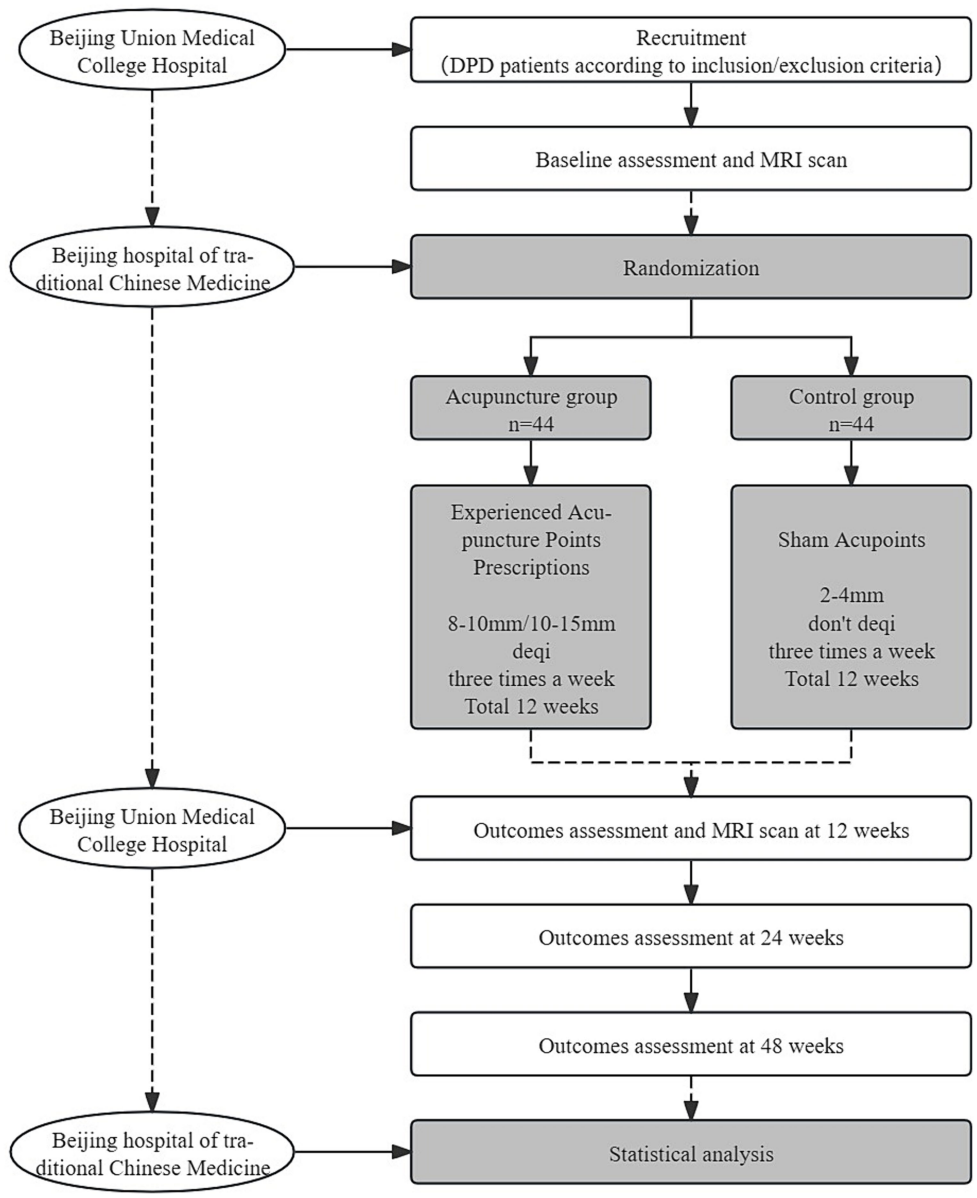
Flow chart.

This trial is a 1:1 randomized, single-blind, and placebo-controlled study with two parallel groups, involving patients with early PD with mild to moderate depression. The study is designed to evaluate the effect and neural mechanism of acupuncture treatment on depression and motor symptoms after a 12-week intervention period. As shown in Figure 1, depression assessments and MRI scans will be performed immediately after baseline and intervention completion. The enrollment schedules, interventions, and assessments are summarized in Table 1.

**Table 1:** Study period.

Items	Screening	Baseline	Treatment	Follow-up	
Timepoint	Week 1	Week 0	Week 12	Week 24	Week 48
Enrolment					
Eligibility screen	X				
Informed consent	X				
Intervention					
Acupuncture (*n* = 44)		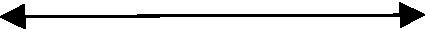			
Sham acupuncture (*n* = 44)		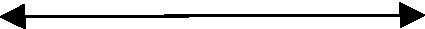			
fMRI scan					
Acupuncture		X	X		
Sham acupuncture		X	X		
Assessment					
HAMD-17		X	X	X	X
UPDRS-III		X	X	X	X
QIDS-SR		X	X	X	X
PDQ-39		X	X	X	X
H-Y		X			X

### Participants

The methodology framework for this project is as follows: the study will be conducted in Dongcheng District, Beijing. The patient with DPD will be recruited from the outpatient department of the Acupuncture Center at Beijing Hospital of Traditional Chinese Medicine and the outpatient department of Neurology of Peking Union Medical College Hospital. To improve residents’ understanding of the trial, a popular science lecture on acupuncture for PD will be held on 11 April (World Parkinson’s Day). Additionally, posters will be displayed at the outpatient clinic of the Acupuncture Center at Beijing Hospital of Traditional Chinese Medicine. Furthermore, the trial advertisement will be posted on the official WeChat account of Beijing Hospital of Traditional Chinese Medicine. Patients interested in the clinical trial will be screened at Beijing Union Medical College Hospital using the inclusion/exclusion criteria. All patients who meet the inclusion criteria will receive an informed consent form that outlines the study objectives, design, procedures, benefits, and risks. Before the commencement of study procedures, subjects will be required to provide written informed consent. The diagnosis of DPD will be conducted following the diagnostic criteria for DPD outlined in the Guidelines for the Diagnosis and Treatment of Depression, Anxiety, and Psychotic Disorders in PD. The Parkinson’s Specialized Clinic at the Department of Neurology of Peking Union Medical College Hospital will assess the level of depression using the 17-item Hamilton Depression Rating Scale (HAMD-17 score >7 and ≤24). The Hoehn–Yahr Staging Scale is employed to evaluate the severity of PD (H–Y score ≤2.5), while the Mini-Mental State Examination (MMSE) is used to assess language and cognitive abilities (MMSE score >20). A Peking Union Medical College Hospital neurologist will fix a drug treatment plan.

### Inclusion criteria

- The patients met the diagnostic criteria of DPD in “Guidelines for the Diagnosis and Treatment of Depression, Anxiety and Psychotic Disorders in Parkinson’s Disease” (31).
- Age range of 50 to 75 years old.
- According to the evaluation by the PD-specific outpatient department of the project cooperation unit, the patient has received standardized medication for at least 3 months, has a fixed anti-PD drug treatment plan, and has no drug adjustment plan for the next 3 months.
- H–Y staging ≤2.5 levels (the guidelines for the diagnosis of PD in China define patients with H–Y stage ≤2.5 as early PD patients) (32).
- Hamilton Depression Rating Scale (HAMD-17) >7 points, and ≤24 points.
- The patients have not received acupuncture treatment for DPD or PD in the past 3 months.
- No language and intellectual disabilities Mini-Mental State Examination (MMSE), able to complete DPD-related scales assessments.
- Participating voluntarily in the study and have signed informed consent to treatment.

### Exclusion criteria

- Patients with secondary Parkinson’s syndrome or Parkinson’s superposition syndrome.
- Patients who are currently taking or have taken traditional antidepressants in the past 2 weeks, such as selective serotonin and norepinephrine reuptake inhibitors (venlafaxine, etc.), selective serotonin reuptake inhibitors (sertraline, paroxetine, fluoxetine, citalopram, etc.), tricyclic antidepressants (amitriptyline, etc.).
- Patients undergoing neurosurgical surgery (deep brain stimulation), psychological therapy, or repeated transcranial magnetic stimulation therapy.
- Patients with serious internal medical conditions such as heart, liver, kidney, hematopoietic system disorders malignant tumors, as well as mental disorders.
- Patients are unable to undergo an fMRI scan for other reasons.

### Randomization and blinding

All DPD participants will be randomly assigned in a 1:1 ratio to one of two groups: the control group and the acupuncture group. Random numbers will be generated by an independent researcher using PROC PLAN in SAS 9.2 (SAS Institute, Cary, NC, United States). The random numbers will be sealed in opaque envelopes by an independent research assistant. Once the random allocation principle is accepted, participants will randomly select an opaque envelope to receive an allocation serial number. The number will then be documented on a case report form (CRF) by a research assistant specifically assigned to this task. To preserve blinding, the acupuncturist, researchers, assessors, and statistical analysts will be kept separate throughout the study. Because of the specific nature of acupuncture treatment, it is not possible to blind the acupuncturists during the treatment. Before the study begins, acupuncturists will be trained uniformly. They will be asked not to disclose the grouping to their patients.

### Interventions

The treatment will receive 36 acupuncture treatments (three times a week) within 12 weeks. Hwato brand disposable acupuncture needles (0.30 × 25 mm or 0.30 × 40 mm in size) will be used.

**Figure 2:**
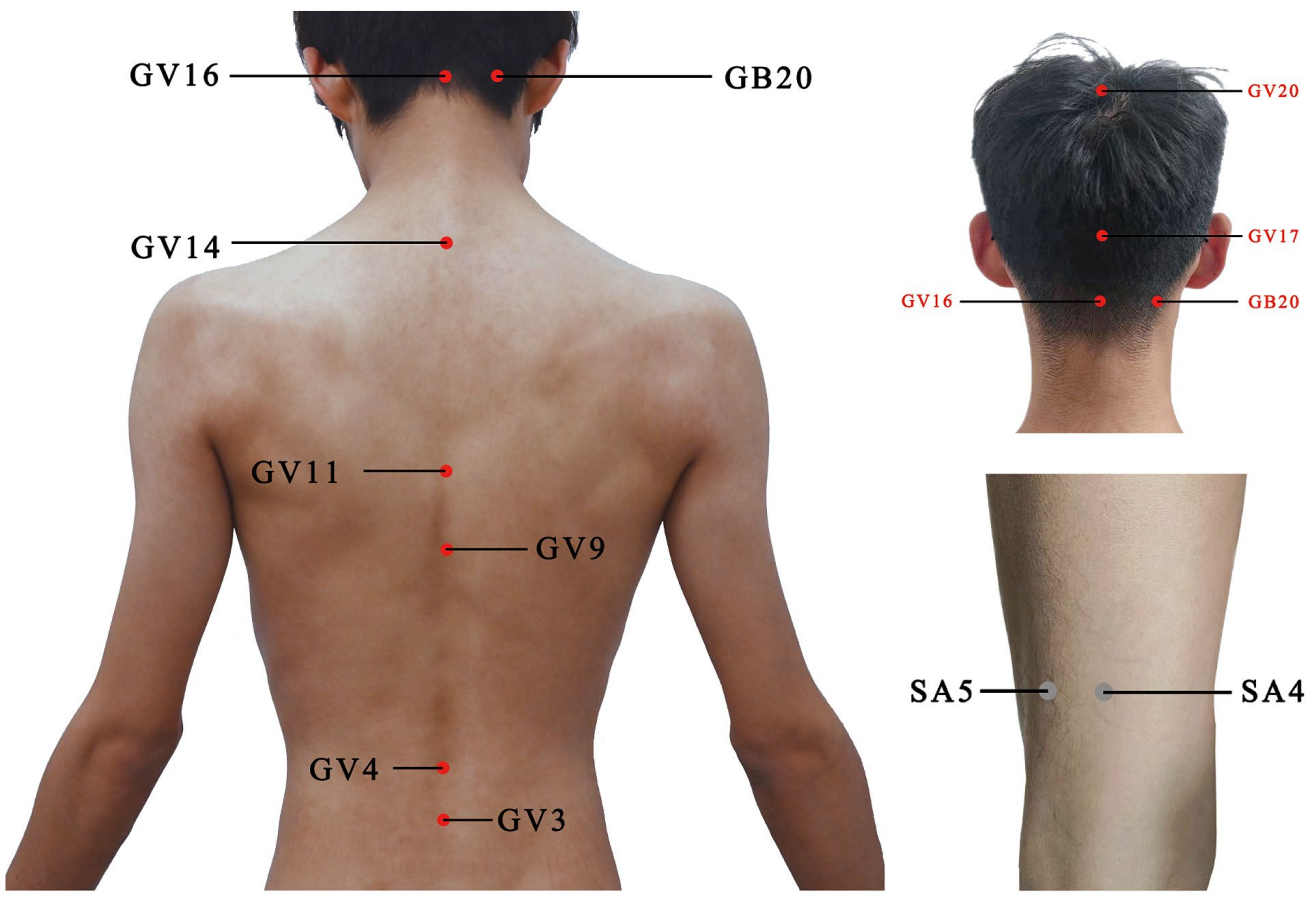
Location of acupoints or sham acupoints in the trial. GV20, Baihui; GV17, Naohu; GV16, Fengfu; GB20, Fengchi; GV14, Dazhui; GV11, Shendao; GV9, Zhiyang; GV4, Mingmen; GV3, Yaoyangguan; SA, sham acupoint.

For the acupuncture group, acupuncture needles will be inserted into acupoints Baihui (GV20), Naohu (GV17), Fengfu (GV16), bilateral Fengchi (GB20), Dazhui (GV14), Shendao (GV11), Zhiyang (GV9), Mingmen (GV4), and Yaoyangguan (GV3) (Figure 2; Table 2). The acupuncture procedure is as follows: the patient is placed in a prone position and undergoes routine skin disinfection, needles (size 0.30 × 25 mm) will be inserted obliquely into Baihui (GV20), Naohu (GV17), and vertically into Fengfu (GV16), bilateral Fengchi (GB20) with a depth of 8–10 mm. After eliciting deqi, the needles will be used for twirling-rotating manipulation. The needles (size 0.30 × 40 mm) will be inserted vertically into Dazhui (GV14), Shendao (GV11), Zhiyang (GV9), Mingmen (GV4), and Yaoyangguan (GV3) with a depth of 10–15 mm. After eliciting deqi, the needles will also be used for twirling-rotating manipulation. Every acupoint requires a 30 s needle manipulation and a 30 min needle retention.

For the control group, we chose the sham acupoint method. The reason why we choose sham acupoint therapy instead of non-invasive therapy is that Chinese patients generally have acupuncture and moxibustion treatment experience, and DPD patients often have strong anxiety and distrust of people and things around them.

It is recommended that sham acupoints be located in areas that are not in proximity to effective acupoints for the treatment of PD and depression. The head acupoints are associated with the central nervous system and are located where meridians are densely distributed to minimize the physiological effects. The sham acupoints were selected as 1 cm on each side of the midpoint of the line connecting Xiaoluo (SJ12) and Naohui (SJ13), 1 cm lateral to the midpoint of the line connecting Naohui (SJ13) and Jianliao (SJ14), and 1 cm on each side of the midpoint of the line connecting Yinmen (BL37) and Fuxi (BL38) (Table 3). After routine disinfection of the skin, needles that are 25 mm long and 0.18 mm in diameter will be oblique needling into sham acupoints at a depth of 2–4 mm. Do not have deqi and lifting, inserting, twisting, and turning. The needles will be kept in place for 30 min.

**Table 2:** Location of acupoints used in the acupuncture group.

	Acupoints	Location	Angle	Depth
1	Baihui (GV20)	5 cun directly above the midpoint of the anterior hairline, or at the midpoint of the line connecting the apexes of the two auricles	45°	8–10 mm
2	Naohu (GV17)	At the top of the head, there is a depression located at the upper edge of the external occipital protuberance	45°	8–10 mm
3	Fengfu (GV16)	On the nape, directly below the external occipital protuberance, lies a depression between the trapezius muscles on both sides	90°	10–15 mm
4	Fengchi (GB20)	On the nape, below the occiput, at the depression between the upper portions of the sternocleidomastoid and trapezius muscles	90°	10–15 mm
5	Dazhui (GV14)	On the midline of the back of the neck, at the depression below the spinous process of the 7th cervical vertebra	90°	10–15 mm
6	Shendao (GV11)	On the midline of the back, at the depression below the spinous process of the 5th lumbar vertebra	90°	10–15 mm
7	Zhiyang (GV9)	On the midline of the back, at the depression below the spinous process of the 7th thoracic vertebra	90°	10–15 mm
8	Mingmen (GV4)	On the midline of the waist, at the depression below the spinous process of the 2nd lumbar vertebra	90°	10–15 mm
9	Yaoyangguan (GV3)	On the midline of the back, at the depression below the spinous process of the 4th lumbar vertebra.	90°	10–15 mm

**Table 3:** Location of sham acupoints used in the control group.

	Sham acupoints	Location
1	Sham acupoints 1	1 cm to the right of the midpoint of the line connecting Xiaoluo (SJ12) and Naohui (SJ13) points
2	Sham acupoints 2	1 cm to the left of the midpoint of the line connecting Xiaoluo (SJ12) and Naohui (SJ13) points
3	Sham acupoints 3	1 cm to the lateral to the midpoint of the line connecting Naohui (SJ13) and Jianliao (SJ14)
4	Sham acupoints 4	1 cm to the right of the midpoint of the line connecting Yinmen (BL37) and Fuxi (BL38)
5	Sham acupoints 5	1 cm to the left of the midpoint of the line connecting Yinmen (BL37) and Fuxi (BL38)

### Sample size calculation

In this study, the sample size was calculated using G*Power 3.1 software, and the HAMD-17 response rate was selected as the primary outcome. We also designed a pre-trial. The results of our pre-trial study showed that the response rate of DPD in the acupuncture group was 92%. When conducting a one-sided significance test, the ratio of the two groups is 1:1, and the setting of *α* = 0.05, *β* = 0.80. The calculated preliminary sample size is 78 patients. Considering a dropout rate of 10%, and a corrected sample size of 87 cases. Ultimately, 88 patients will be included, with 44 patients in each group.

### Outcome measures

#### Hamilton Depression Scale-17

Hamilton Depression Scale-17 (HAMD-17) is recommended as the primary endpoint for depression in clinical trials (33). HAMD-17 will evaluate changes in depression levels from baseline to the end of the follow-up.

#### MDS Unified-Parkinson Disease Rating Scale-III

The MDS Unified-Parkinson Disease Rating Scale-III (UPDRS-III), revised by the International Parkinson and Movement Disorder Society, is the most commonly used evaluation scale for PD in clinical and scientific research settings (34, 35). The UPDRS-III will evaluate changes in motor symptoms by counting tremor and non-tremor symptoms of PD.

#### Quick Inventory of Depressive Symptomatology-Self Report

The Quick Inventory of Depressive Symptomatology-Self Report (QIDS-SR) scale is a simple and feasible depression screening scale. It exhibits high sensitivity, specificity, and clinical practicality (36, 37). The QIDS-SR will assist in evaluating changes in depression levels from baseline to the conclusion of the follow-up period.

#### Parkinson’s Disease Patient Quality of Life Questionnaire-39 (PDQ-39)

The PDQ-39 can assess the QoL for Chinese patients with PD, regardless of whether they have cognitive impairment (38, 39). The PDQ-39 will be used to evaluate changes in QoL from the baseline to the end of the follow-up.

#### MRI data

Patients will undergo cranial magnetic resonance imaging at baseline and after treatment. The MRI scan will be conducted using a 3.0 Tesla superconducting magnet (Siemens MAGNETOM Skyra 3.0 T) at the Beijing Hospital of Traditional Chinese Medicine Affiliated with Capital Medical University. The parameters of the sequences used are as follows: Slices = 300, TR = 2,000 ms, TE = 30 ms, Slice thickness = 4.0 mm, FoV read = 200 × 200 mm^2^, Flip angle = 90°, Voxel size = 3.1 × 3.1 × 4.0 mm, Base resolution = 64, Phase resolution = 100%, TA = 10.06 min.

##### Primary indicator

The response rate (The response rate, defined as the proportion of total scores that decreased by at least 50% compared with baseline statistics) of HAMD-17 (at week 12).

##### Secondary indicator

The change from baseline in resting-state fMRI (at week 12). The response rate of HAMD-17 (at weeks 24, and 48). The changes from baseline in UPDRS-III and PDQ-39 (at weeks 12, 24, and 48). The response rate of QIDS-SR, and the change from baseline in PDQ-39 (at weeks 12, 24, and 48). The proportion of patients with progression≥0.5 grades compared to the baseline of H–Y staging (at week 48).

### Safety assessments

Any adverse effects (AEs) happening throughout the study period, whether reported spontaneously by the participants or observed by the researchers, will be recorded and categorized as needling-related AEs. Common adverse events associated with acupuncture include persistent post-needling pain, dizziness, subcutaneous hematoma, infection, etc. Serious adverse events will be reported and handled according to relevant regulations within 24 h.

### Data management and quality control

At baseline, information on the patient’s age, gender, occupation, education level, dominant hand, medical history, etc. We will arrange for patients who meet the inclusion criteria to undergo questionnaire evaluation in a quiet room with no unrelated personnel, followed by an fMRI examination. All fMRI will be conducted using the same 3.0 T MRI machine.

The case report form (CRF) that has been approved on ethical grounds will be used to collect clinical data on each enrolled patient. The data from the paper CRF forms will be entered into a Microsoft Excel spreadsheet independently by two researcher assistants. A comparison of the data entry results between the two research assistants will be conducted to identify any inconsistencies. Any discrepancies in the electronic data will be validated by a third independent research assistant who will review the paper data. The electronic data will be analyzed using SPSS 20.0 software (IBM Corporation, Armonk, NY, United States).

### Statistical analysis

#### Clinical data analysis

Statistical analysis will be conducted utilizing SPSS 20.0 (IBM Corporation, Armonk, NY, United States), a software package to analyze statistical data. Statistical significance was determined at the 0.05 level (two-tailed). The intention-to-treat (ITT) analysis will be employed as the primary analysis for all patients, and missing data were filled in using the last observation carried forward (LOCF). A supplementary analysis will be conducted using the adherence protocol analysis (PP).

The categorical data will be described using frequencies and percentages. The proportion of patients with progression ≥0.5 grades compared to the baseline of H–Y staging, and adverse events were analyzed using the chi-squared test. The initial step in the analysis of continuous data is to assess their normality. The data are described using mean ± standard deviation or median (interquartile range). Within-group comparisons are conducted using either paired *t*-tests or Wilcoxon signed-rank tests, while between-group comparisons are carried out using independent samples *t*-tests or Mann–Whitney *U* tests. HAMD-17, QIDS-SR, UPDRS-III, and PDQ-39 were analyzed using either repeated-measures ANOVA or a generalized linear mixed model based on repeated measurements.

#### Image data analysis

All scans will undergo qualitative review by two radiologists to screen for potential brain lesions or structural abnormalities. The data processing is based on the MATLAB 2014a platform and is carried out using DPARSF V2.3. Using DPARSF V2.3, SPM12, and REST V1.8 for seed-based functional connectivity analysis. Using FSL 4.1 MELODIC ICA for independent component analysis. Using REST V1.8 for the Regional homogeneity method (ReHo). Utilizing DPARSF V2.3, SPM12, and REST V1.8 for Fractional Amplitude of low-frequency fluctuation (fALFF).

### Progress of research

The subject is currently recruiting patients continuously, and patients have been enrolled for regular interventions and follow-ups as per the trial design.

## Discussion

The principal objectives of this study are to evaluate the clinical efficacy and safety of acupuncture in the treatment of DPD and to investigate its mechanisms of neural regulation. The results of these studies will contribute to the establishment of a therapeutic program for effective and safe acupuncture intervention in DPD.

Depression is a common occurrence in early PD and persists throughout all stages, significantly impacting patients’ quality of life (40). The development of new therapeutic approaches to treat DPD is essential to improve the quality of life of DPD patients (41). This study was conducted following the academic experience of a renowned Chinese physician, Prof. Cheng Haiying, in treating PD. This study was a randomized, evaluator-blinded, sham acupuncture placebo-controlled trial to assess the efficacy and safety of acupuncture intervention for early mild-to-moderate DPD.

The present study has several innovative strengths. First, to the best of our knowledge, this study represents the first randomized controlled trial of acupuncture treatment for patients with early PD (H–Y staging ≤ grade 2.5) and mild-to-moderate depression, based on the internationally recognized Hoehn–Yahr staging criteria for PD. This study addresses the shortcomings of previous acupuncture research by separating DPD patients based on the severity of their condition. It investigates the potential benefits of early acupuncture intervention in DPD to determine if it can effectively delay the progression of PD during mid to long-term follow-up.

Secondly, this study selected scale and questionnaires and a combination of brain fMRI technology as the efficacy evaluation indicators. This study included fMRI data collection to explore the objective mechanism of acupuncture for early Parkinson’s disease with mild to moderate depression. Recent neuroimaging studies have begun to reveal the central mechanism of DPD (42–44), but the mechanism of acupuncture interventions to regulate the central nervous system remains is still unclear. The present study provides a foundation for future research on the central mechanisms of acupuncture treatment.

One potential limitation of this study is that, due to the practicalities of acupuncture treatment, blinding for acupuncturists cannot be implemented. A further limitation of this study is that we were unable to control medication use. To address this limitation, we had a professional physician conduct a systematic specialty assessment before enrolling patients. It was anticipated that no routine medication adjustments would be necessary for the duration of the next 3 months.
